# Chemotherapy-induced neuropathy (CIN) persists chronically for most individuals with breast cancer who develop the condition: A survey study

**DOI:** 10.21203/rs.3.rs-8594232/v1

**Published:** 2026-01-28

**Authors:** Lise Worthen-Chaudhari, Maryam B. Lustberg, Bhillie D. Luciani, Margaret E. Gatti-Mays, Sagar D. Sardesai, Ashley P. Davenport, Patrick M. Schnell

**Affiliations:** The Ohio State University; Yale University; The Ohio State University; The Ohio State University; The Ohio State University; The Ohio State University; The Ohio State University

## Abstract

**Purpose:**

A majority of individuals treated with taxane agents for breast cancer (BC) develop chemotherapy-induced neuropathy (CIN) yet questions remain regarding long-term prognosis of this neurologic complication. We sought to characterize CIN chronicity in terms of persistence rate, predictors, and neuromotor dysfunction.

**Methods:**

We surveyed individuals with BC who developed CIN during taxane exposure at least 3 months prior. We calculated the proportion of survey respondents with/without chronic CIN symptoms and performed a sensitivity analysis, computing the largest and smallest possible rate of CIN chronicity given the number of non-respondents. Additionally, we analyzed predictors of chronicity (e.g., age, hgA1c level) and characterized the proportion of individuals with chronic sensory symptoms who also demonstrated neuromotor dysfunction.

**Results:**

We surveyed two hundred and fifty-eight individuals (99.2% female). Mean(SD) age = 60.5(11.1); time since last taxane exposure = 3.25(2.38) years; and A1C = 5.64(0.607). Persistent CIN sensory symptoms were reported by 84.9% [95% CI 79.5%, 90.3%]. Accounting for both the non-response sensitivity analysis and statistical uncertainty, we calculate bounds for the CIN persistence rate to be no lower than 50.5% and no higher than 93.6%. Variables predictive of CIN chronicity included high A1C (p = 0.037). Variables not predictive included total chemotherapy dose (p = 0.8); race (white vs non-white, p = 0.4); age (p = 0.09); time since last taxane exposure < 6 years (p = 0.051). Among those with chronic CIN, 85% demonstrated quantifiable neuromotor dysfunction.

**Conclusion:**

CIN symptoms persist chronically for most individuals (~ 85%) with breast cancer who develop the condition during taxane treatment. Novel interventions are needed to alleviate sensory symptoms and associated neuromotor dysfunction.

## Introduction

Individuals who have undergone cancer treatment report chemotherapy-induced neuropathy (CIN) as one of their highest, unmet priorities within survivorship, yet questions remain about scope of the problem among breast cancer survivors (Lustberg et al., 2023). It is commonly summarized that CIN resolves spontaneously within 3–6 months after conclusion of neurotoxic chemotherapy treatment for the majority of individuals who develop the condition from any type of cancer treatment (Maihöfner et al., 2021; Miltenburg & Boogerd, 2014; Seretny et al., 2014). However, a growing body of evidence indicates that CIN does not resolve for the majority of individuals with breast cancer who develop the condition (Bao et al., 2016; Cavaletti et al., 2015; Lavoie Smith et al., 2013; Mo et al., 2022; Seretny et al., 2014; Winters-Stone et al., 2017). If our common understanding that CIN spontaneously resolves for most individuals with breast cancer is incorrect then the need to prevent and rehabilitate CIN is more stark than formerly understood for this population. We performed this survey study to calculate CIN chronicity rate among individuals with breast cancer. Additionally, we sought to illuminate predictors and neuromotor consequences of chronic CIN in this population.

Chemotherapy-induced neuropathy is a neurologic complication caused by exposure to cytotoxic chemotherapy drugs (e.g. paclitaxel, docetaxel) (Hu et al., 2019; Lavoie Smith et al., 2013). Once it develops, CIN can lead to discontinuation of treatment in the short-term (Song et al., 2017; Winters-Stone et al., 2023) and functional concerns in the long-term (Loprinzi et al., 2020; Lustberg et al., 2023; Song et al., 2017). Symptoms present as pain, numbness, tingling, burning (Tanay et al., 2017), and/or temperature sensitivity in the hands and feet (Jaggi & Singh, 2012; Li et al., 2021; Lustberg et al., 2023; Mezzanotte et al., 2022). In addition to uncomfortable sensory symptoms, CIN causes functional neuromotor deficits (Fino et al., 2019; Monfort et al., 2016, 2017, 2019) including increased risk of falling (Bao et al., 2016; Huang, 2019; Wechsler & Wood, 2021).

Indirect evidence contradicts the belief that CIN resolves spontaneously among the majority of individuals with BC who develop the condition (Bao et al., 2016; Osmani et al., 2012; Postma et al., 1995; Winters-Stone et al., 2017). For example, Bao et al (2016) found that 51.2% of 296 post-menopausal women with breast cancer reported persistent and chronic CIN more than 5 years after discontinuing chemotherapy. Other datasets also suggest that CIN persists for a majority of those who develop it but do not calculate the rate of chronicity among individuals with BC, specifically (Osmani et al., 2012; Postma et al., 1995; Winters-Stone et al., 2017).

We postulate that spontaneous resolution is atypical among individuals with breast cancer and hypothesize that a majority (> 50%) of individuals with BC who develop CIN symptoms during taxane-based treatment go on to experience the condition chronically (i.e., > 3 months post taxane-based chemotherapy discontinuation). Additionally, to expand understanding of predictors and functional consequences of CIN among individuals with BC, we analyzed predictors of chronicity (e.g., age, HgA1C level, time since chemotherapy completion) and quantified postural control dysfunction in a subset of survey respondents with chronic CIN. This study was performed as a secondary analysis of data collected for clinical trial NCT05114005 among individuals with breast cancer and CIN diagnoses (R21-AG068831).

## Methods

This research protocol was approved by the Ohio State University (OSU) Institutional Review Board (IRB# 2015C0090). The full study protocol is described in Lantis et al. (Lantis et al., 2023) and was registered in ClinicalTrials.gov (NCT05114005); the present analysis examines recruitment and screening data from NCT05114005. Participant identification was comprised of three steps: electronic medical record screenings for taxane exposure plus documented patient report of CIN, contact and survey of CIN prevalence among eligible clients, and postural control data collections among an interested subset of survey respondents.

### Electronic Medical Record (EMR) Screening:

**Figure 1:**
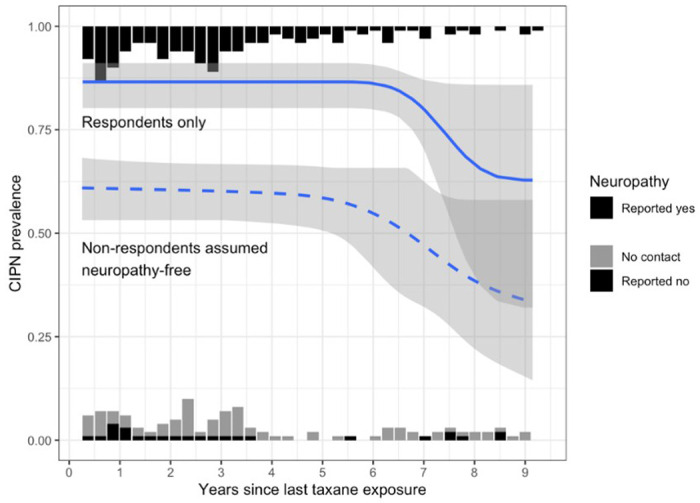
Estimated prevalence of persistent CIN over time among patients exposed to taxanes at least three months prior and having reported neuropathy development during taxol treatment. Curves and shaded regions indicate estimated prevalence and 95% confidence intervals from logistic regression with monotonically decreasing splines. Bars indicate counts of patients with (top) and without (bottom) neuropathy, not related to vertical axis scale. Changes in prevalence over time are not statistically significant (respondents only p =0.06).

As part of the study protocol, we queried patient records within OSU’s academic medical system to identify eligible participants living with breast cancer diagnosis who reported development of CIN symptoms during taxane-based chemotherapy exposure, were ≥ 40 years of age. We included any gender and individuals as young as 40 years of age because recent literature indicates that neither younger age nor gender are protective against development of CIN (Argyriou et al., 2025). From these records, we identified clients potentially eligible for participation in NCT05114005 who were treated within our medical center between the dates of March 11, 2014 and February 9, 2023. We used semantic search (i.e., text-based search of the record), to determine if the client reported neuropathy to a provider at any point during treatment or in the weeks following chemotherapy discontinuation (Fig. 1). Lastly, we used semantic search to exclude those with uncontrolled diabetes (hbA1c > 8.0) or neurologic disorder other than CIN (e.g. vestibular disorder, Parkinson Disease).

### Survey:

We sought permission to contact clients who were deemed potentially eligible for NCT05114005 from their treating oncologist. To focus contact efforts among clients with whom providers were more recently familiar, we prioritized individuals who were between 0.25 and 6 years post chemotherapy completion, however, we included individuals up to 9 years post chemotherapy completion. Those cleared by the treating oncologist for contact were aggregated in a list sorted by medical record number and entered as record items within a secure Research Electronic Data Capture (REDCap) database (Nashville, TN). We assigned a pre-screen number in REDCap per potentially eligible individual and systematically attempted contact to determine (a) did CIN symptoms persist and (b) was the individual interested in an intervention trial targeting the persistent symptoms until recruitment for NCT05114005 was complete. Among those individuals who responded to our attempted contact and agreed to survey participation, we asked if CIN persisted as follows: “Your oncologist indicated that you experienced neuropathy symptoms during your treatment. Are you still experiencing neuropathy?” We documented contact results in the REDCap database.

### Statistical Analysis

For our primary estimate of rate of chronicity, we computed the proportion of eligible participants who reported persistent CIN symptoms among those who responded to our survey. This approach assumes that responders were representative of the full eligible population (including non-responders) with respect to neuropathy. As a sensitivity analysis, we also computed proportions, assuming that all eligible non-respondents had neuropathy (i.e., maximum estimate) or did not have neuropathy (i.e., minimum estimate). Confidence intervals were computed via binomial test. Characteristics are expressed as mean (SD) and/or as median [min, max]. Prevalence over time was modeled using monotonically decreasing spline logistic regression.

### Neuromotor dysfunction among those with persistent sensory symptons:

If contact was successful and responders reported persistent CIN symptoms at the time of contact, we invited responders to undergo an in-person screening of postural control (Reed et al., 2020; Worthen-Chaudhari et al., 2018). Briefly, screening involved participants standing bilaterally and quietly on a balance plate (Bertec Corp, Columbus, OH) for 30 seconds with eyes closed (QEC). Center of pressure (COP) variables of interest were calculated per Worthen-Chaudhari et al. (2018) (Worthen-Chaudhari et al., 2018), Prieto et al. (1996) (Prieto et al., 1996), and Roerdink et al. (2011) (Roerdink et al., 2011) using a custom MATLAB program (MathWorks, Natick, MA).

We analyzed the number of respondents with CIN who demonstrated postural control function outside of the norm. As previously defined (Lantis et al., 2023), normative thresholds were estimated as outside of the 70% confidence interval (CI) of healthy, age-equivalent values (Worthen-Chaudhari et al., 2018) in variables that have proven to predict future fall risk (Maki et al., 1994; Worthen-Chaudhari et al., 2018) (i.e., COP ellipse area (COPa), medial-lateral variability (RMSml), medial-lateral velocity (COPvml)) and the estimated 95% CI in terms of COP complexity (SEI), a non-linear measure that has more recently been associated with neurologic challenge (Donker et al., 2007, 2008; Quatman-Yates et al., 2015; Roerdink et al., 2011; Stins et al., 2009). Those that fell outside of postural control norms in any of the four measures (i.e., COPa, RMSml, COPvml, SEI) were deemed to have motor dysfunction associated with persistent, self-reported CIN symptoms.

## Results

We identified 1966 records that potentially met eligibility criteria. Six hundred and fourteen clients were excluded due to deceased status, A1C levels > 8.0, or additional neural diagnosis such as vestibular disorder. The remaining 1352 records were categorized as having reported development of CIN to a provider and receiving a CIN diagnosis (748; 55.3%) or no report of CIN development during chemotherapy exposure (604; 44.7%). Of the individuals diagnosed with CIN at the time of treatment, we attempted contact with n = 258 for the purpose of surveying (a) current presence/absence of CIN symptoms and (b) interest in research-based nonpharmacologic options addressing these symptoms. One hundred and forty-six survey respondents confirmed that CIN persisted, while 26 reported that CIN had resolved completed or “did not bother them anymore”. Eighty-six individuals could not be contacted or declined to be surveyed.

### Survivor characteristics

See Table 1 for details including mean(SD) and median[minimum, maximum] calculations per category. Among the overall cohort with whom we attempted contact before the survey ended (i.e., when recruitment goals for NCT05114005 were achieved), mean (SD) age was 60.5 (11.1) years; A1c level was 5.64 (0.61); and time since last taxane exposure was 3.25 (2.38) years. A majority were white (82.6%) and female (99.2%). Regarding taxane treatment, 41.9% were exposed to docetaxel only; 55.4% were exposed to paclitaxel only; and 2.7% were exposed to both docetaxel and paclitaxel. Among those receiving docetaxel only, mean (SD) overall exposure was 352 (97) mg/m^2^; 20.2% received a dose reduction; and 0% received a dose increase. Of those receiving paclitaxel only, mean summed dosage was 721 (232) mg/m^2^; 29.8% received a dose reduction; and 0.4% received a dose increase. Five individuals (1.9%) switched chemotherapy agents from docetaxel to paclitaxel while 2 switched from paclitaxel to docetaxel (0.8%). Table 1 further details medication dose and other characteristics reported by neuropathy subgroup as well as for the entire cohort.

Finally, we analyzed differences between the 146 respondents with and 26 without chronic CIN. Only higher A1C levels predicted persistence of CIN for this cohort of individuals with BC who reported CIN during treatment (p = 0.037). Additionally, when considering those who received docetaxel only or paclitaxel only, with no combination of the two agents, those receiving docetaxel only showed a trend toward more frequent CIN persistence when compared to those receiving paclitaxel only (p = 0.077). No other factors predicted persistence of CIN including race, ethnicity, time since last taxol, age, or total taxane dose.

#### Persistent sensory dysfunction estimate:

CIN persisted for 146 (84.9%) of those who responded to our survey (responders) (95% confidence interval for population 79.5% to 90.3%). Our sensitivity analysis estimated a minimum and maximum possible count as follows: assuming all non-responders experienced resolution of CIN, then the minimum possible count of survivors with chronic sensorimotor neuropathy symptoms is 146 of 258 (56.6%; 95% CI 50.5% to 62.7%) and assuming all non-responders had persistent CIN, then the maximum possible count of survivors with chronic sensorimotor neuropathy symptoms is 232 of 258 (89.9%; 95% CI 86.2% to 93.6%). Accounting for both the non-response sensitivity analysis and statistical uncertainty, we calculate bounds for the CIN persistence rate to be 50.5% to 93.6%. Figure 1 depicts the estimated prevalence of persistent CIN as a function of years since last taxane exposure, based on respondents only and separately assuming that all non-respondents were neuropathy-free. Overall, changes in prevalence of persistence over time are not significant (respondents only p = 0.06, assuming all non-respondents are neuropathy-free p = 0.11). More specifically, in the first 5–6 years post treatment completion, CIN persistence does not appear to decrease with time. Beyond 6 years post-treatment there is insufficient data for reliable conclusions.

#### Persistent neuromotor dysfunction estimate

Among 146 respondents with chronic CIN, 59 performed in-person postural control screening. Balance measures were outside of normal healthy parameters for 86.4% (n = 51) and within normal parameters for 13.5% (n = 8). Postural sway values for those outside of normal healthy parameters are reported elsewhere (Lantis et al., 2023).

## Discussion

Chronic CIN persisted for 84.9% [50.5%, 93.6%] of individuals with BC who were diagnosed with the condition during taxane-based chemotherapy treatment. Mean time since last taxane exposure for this cohort was more than 3 years. This result substantiates our hypothesis that CIN persists chronically, for many years, for most individuals with breast cancer who develop it. Time since last taxane exposure was not predictive of chronic CIN through at least 6 years post chemotherapy completions. However, A1C levels at the time of survey were predictive such that a higher A1C level was associated with increased risk of developing persistent CIN. Addi

In addition to clarifying the rate of CIN persistence as higher than previously believed among individuals with BC, this study provides novel insights into the functional deficits associated with persistent CIN in this population. We calculated the rate of postural control deficits to be 85% [80%, 90%] among those who presented for in-person biomechanical testing. Therefore, of the ~ 85% of individuals in this cohort who reported persistent CIN, approximately 85% demonstrated quantifiable balance deficits in addition to patient-reported sensory symptoms. Because postural control dysfunction predicts increased fall risk, independent of sensory symptoms (Maki et al., 1994; Worthen-Chaudhari et al., 2018), this high rate of postural control dysfunction represents a potential neuromotor mechanism underlying the increased fall risk of individuals with CIN (Bao et al., 2016; Winters-Stone et al., 2023). Postural control deficits can be added to known functional consequences of CIN, in addition to known deficits in dual-task performance (Worthen-Chaudhari et al., 2024), gait characteristics (i.e., shorter and slower steps), chair-stand test, and short physical performance battery (Winters-Stone et al., 2017). There remains a need for more prospective studies characterizing the nature, scope, and mechanisms underlying functional deficits resulting from CIN. These should measure CIN symptoms using mixed-methods that combine patient-reported outcomes of sensation with quantitatively measured function.

There are at least two ways in which the belief that CIN resolves spontaneously for most individuals with breast cancer may have developed. Firstly, very little literature has focused on breast cancer, specifically. Instead, most original research of CIN resolution rates after treatment tends to group CIN from different types of cancers together. For instance, a meta-analysis and systematic review by Seretny et al. (2014) pooled data from 4179 individuals who received chemotherapy of any type for any cancer diagnosis. In this meta-analysis, approximately half of those diagnosed with CIN reported that the condition improved between 3- and 6-months post chemotherapy completion. It is possible that results were assumed to generalize to individuals with BC.

A second way that the belief of spontaneous CIN resolution may have developed is through misinterpretation of the original data. We found that two original data sets, from Postma et al (1995) and Osmani et al (2012), are cited to substantiate this interpretation. However, neither dataset supports the idea of spontaneous, complete resolution within 3 months.

Postma et al. (1995) followed 7 individuals with BC; all 7 developed symptoms within their first course of high-dose paclitaxel. The authors followed 5 of these individuals for 9 months post chemotherapy termination, finding that 3 of 5 reported complete resolution of pain; all 5 reported changing CIN sensation in the 9 months following chemotherapy termination; however, none reported complete resolution of symptoms such as numbness, tingling, or functional deficits. Based on these results, Postma et al. (1995) conclude that CIN is “at least partially reversible” after discontinuation of paclitaxel exposure but the authors do not interpret their results to mean that spontaneous, complete resolution occurs for a majority within 6 months (Postma et al., 1995).

The second dataset cited to substantiate the claim of CIN spontaneous resolution is published in Osmani et al. (2012) “Taxane-based neuropathy has a good long-term prognosis: a 1 to 13 year evaluation”. Within this dataset, only 6 of those diagnosed with CIN, or 14% or those surveyed, reported complete recovery within the 6 months following taxane-based treatment completion. Based on limited relative improvement, reported retrospectively by 86% of those with persistent CIN, the authors conclude that long-term prognosis for complete symptom resolution is good (Osmani et al., 2012) and infer that a majority experience spontaneous resolution. Of note, more than 80% of participants in this study were treated with docetaxel alone, yet results are generalized to all taxane-based CIN diagnoses. Interestingly, a close look at the data in these original reports substantiates our current estimate of CIN persistence among approximately 85%. Osmani et al (2012) report that 86% reported persistence of symptoms, albeit at a reduced severity to initial onset, and only 14% reported complete, spontaneous resolution of CIN. These original studies, cited to support the belief that CIN spontaneously resolves for a majority, ultimately support our current finding of chronic symptom persistence among approximately 85% of individuals with BC who developed CIN during chemotherapy treatment.

### Limitations

While our study provides valuable insights into CIN persistence, limitations should be considered. For instance, our identification of those who reported CIN symptoms during treatment was limited to individuals detected via retrospective semantic search of electronic medical records. Because the text describing CIN in patient charts is not standardized across providers and years, the key terms describing CIN symptoms and diagnosis might have changed over the 9 year period within which we searched and between the different providers who we worked with to complete this survey. Therefore, our results may underestimate the incidence of those who developed CIN during treatment. Conversely, selection bias may have affected who responded to our survey, such that clients who felt better may have responded less frequently to our survey than those who felt worse, skewing estimates toward worse outcomes. Although this possibility is accounted for by our sensitivity analysis, it is important to note. Additionally, this data set is limited in that we confirm persistence of symptoms but lack the ability to confirm consistency of specific symptoms over the period of time between onset and time of survey. Postma et al (1995) provided initial evidence that neuropathic presentation of chronic CIN changes over time such that presence of symptomatology is consistent, but characteristics of symptoms may be dynamic. Additionally, more recent evidence suggests that neuropathic presentation of CIN might change over time, with one case report documenting a short period of resolution between 3 and 6 months post-chemotherapy completion followed by reemergence of tingling and coldness symptoms in the 2–6 years post chemotherapy completion (Papautsky et al.). Among individuals with BC, future research should use a repeated measures design over at least 5 years to characterize the changes in CIN symptoms post completion of chemotherapy.

Despite these limitations, we found that most individuals with BC and CIN – almost 6 in 7 – experience persistent CIN symptoms its that do not spontaneously resolve within 3 months amd that a majority of these individuals also demonstrate neuromotor dysfunction. Given the impact of CIN on wellness in survivorship (Lustberg et al., 2023), there is a need to develop and implement targeted interventions to improve symptoms, function, and quality of life for affected individuals. Our findings reinforce the urgent need for routine long-term follow-up and neurorehabilitation strategies to address the burden of chronic CIN among BC survivors. More research is needed to characterize the dynamics of CIN symptoms through survivorship (Papautsky et al., in press; Postma et al., 1995) and determine best practices to prevent and mitigate this condition that sits at the intersection of oncologic, neurologic, and rehabilitative medicine.

## Supplementary Material

This is a list of supplementary files associated with this preprint. Click to download.

• datapersistencepublic.csv

• cipnpersistencepublicanalysiscode.qmd

## Figures and Tables

**Table 1:** Characteristics of individuals with breast cancer and CIN grouped by survey response including age, time since last taxane exposure, race, ethnicity, A1c level, the taxane-based chemotherapy agent they were exposed to, and the total cumulative dosage of that agent they received.

	Neuropathy Persists (N = 146)	Neuropathy Resolved (N = 26)	No Response (N = 86)	Overall (N = 258)	Persists v Resolved (p value)
Age (Years)					
Mean (SD)	61.8 (10.5)	58.3 (9.40)	58.8 (12.2)	60.5 (11.1)	0.091
Median [Min, Max]	62.0 [40.0, 85.0]	57.5 [42.0, 76.0]	58.0 [40.0, 90.0]	61.0 [40.0, 90.0]	
Time since last Taxol (Years)					0.051
Mean (SD)	3.06 (2.20)	3.23 (2.77)	3.60 (2.55)	3.25 (2.38)	
Median [Min, Max]	2.64 [0.26, 9.14]	2.29 (0.38, 8.46]	3.01 [0.25, 9.10]	2.77 [0.25, 9.14]	
Missing Count (%)	0 (0.0%)	0 (0.0%)	3 (3.5%)	3 (1.2%)	
Cancer stage					
I	62 (42.5%)	12 (46.2%)	29 (33.7%)	103 (39.9%)	
II	61 (41.8%)	7 (26.9%)	38 (44.2%)	106 (41.1%)	
III	17 (11.6%)	6 (23.1%)	15 (17.4%)	38 (14.7%)	
IV	2 (1.4%)	0 (0.0%)	2 (2.3%)	4 (1.6%)	
Metastatic disease	1 (0.7%)	1 (3.8%)	2 (2.3%)	4 (1.6%)	
Missing	3 (2.1%)	0 (0.0%)	0 (0.0%)	3 (1.2%)	
Race					
White	123 (84.2%)	24 (92.3%)	66 (76.7)	213 (82.6%)	0.376^
Black	15 (10.3%)	1 (3.8%)	12 (14.0%)	28 (10.9%)	
Asian/Pacific Islander	1 (0.7%)	0 (0%)	6 (7.0%)	7 (2.7%)	
American Indian / Alaskan	1 (0.7%)	0 (0%)	1 (1.2%)	2 (0.8%)	
Multiple	1 (0.7%)	1 (3.8%)	0 (0.0%)	2 (0.8%)	
Unknown/ Not reported	5 (3.4%)	0 (0%)	1 (1.2%)	6 (2.3%)	
Missing	0 (0.0%)	0 (0%)	0 (0.0%)	0 (0.0%)	
Ethnicity					
Latino / Hispanic	3 (2.1%)	1 (3.8%)	1 (1.2%)	5 (1.9%)	0.484^
Not Latino / Hispanic	143 (97.9%)	25 (96.2%)	84 (97.7%)	252 (97.7%)	
Unknown/ Not reported	0 (0%)	0 (0%)	1 (1.2%)	1 (0.4%)	
Missing	0 (0.0%)	0 (0%)	0 (0%)	0 (0%)	
A1c (%)					
Mean (SD)	5.65 (0.620)	5.40 (0.446)	5.70 (0.621)	5.64 (0.607)	**0.037** *
Median [Min, Max]	5.60 [4.50, 7.70]	5.30 [4.80, 6.50]	5.60 [4.70, 7.80]	5.50 [4.50, 7.80]	
Missing Count (%)	27 (18.5%)	3 (11.5%)	22 (25.6%)	52 (20.2%)	
Taxanes Exposed To					0.077
Docetaxel (Doce)	63 (43.2%)	6 (23.1%)	39 (45.3%)	108 (41.9%)	
Paclitaxel (Pacli)	78 (53.4%)	19 (73.1%)	46 (53.5%)	143 (55.4%)	
Doce + Pacli	5 (3.4%)	1 (3.8%)	1 (1.2%)	7 (2.7%)	
Missing	0 (0.0%)	0 (0.0%)	0 (0.0%)	0 (0.0%)	
Docetaxel Total Exposure (mg/m^2^)					0.491
Mean (SD)	347 (73.5)	366 (117)	365 (132)	352 (97.0)	
Median [Min, Max]	345 [225, 450]	300 [285, 450	375 [195, 960]	345 [195, 960]	
Missing	0	0	3	3 (1.2%)	
Paclitaxel Total Exposure (mg/m^2^)					0.245
Mean (SD)	745 (247)	659 (289)	708 (171)	721 (232)	
Median [Min, Max]	720 [72, 1440]	700 [80, 960]	700 [210, 960]	700 [72, 1440]	
Missing	5	0	1	6 (2.3%)	

^ largest category v. other categories grouped,

* p < 0.05.

## Data Availability

Data are available in supplementary files associated with this manuscript.
